# Facial Nerve Monitoring During Parotidectomy:A Two-Center Retrospective Study

**Published:** 2016-07

**Authors:** Stanislas Ballivet-de Régloix, Julia Grinholtz-Haddad, Olga Maurin, Louise Genestier, Quentin Lisan, Yoann Pons

**Affiliations:** 1 *Department of Otorhinolaryngology – Head and Neck Surgery, Military Training Hospital Percy 101, avenue Henri Barbusse 92140 Clamart, France.*; 2 *Department of Otorhinolaryngology – Head and Neck Surgery, Versailles Hospital Center, André Mignot Hospital 177, rue de Versailles 77157 Le Chesnay cedex, France.*; 3 *Department of Otorhinolaryngology – Head and Neck Surgery, Military Training Hospital Val de Grâce74, boulevard de Port Royal 75005 Paris, France. *

**Keywords:** Facial nerve, Facial paralysis, Intraoperative Monitoring, Parotid gland, Reoperation

## Abstract

**Introduction:**

We present a retrospective two-center study series and discussion of the current literature to assess the benefits of facial nerve monitoring during parotidectomy.

**Materials and Methods:**

From 2007 to 2012, 128 parotidectomies were performed in 125 patients. Of these, 47 procedures were performed without facial nerve monitoring (group 1) and 81 with facial nerve monitoring (group 2). The primary endpoint was the House-Brackmann classification at 1 month and 6 months. Facial palsy was determined when the House-Brackmann grade was 3 or higher.

**Results:**

In group 1, 15 facial palsies were noted; 8 were transient and 7 were definitive. In group 2, 19 facial palsies were noted; 12 were transient and 7 were definitive. At both one and six months after parotidectomy, the rate of facial palsy in reoperation cases was significantly higher in group 1 than in group 2.

**Conclusion:**

Facial nerve monitoring is a simple, effective adjunct method that is available to surgeons to assist with the functional preservation of the facial nerve during parotid surgery. Although it does not improve the facial prognosis in first-line surgery, it does improve the facial prognosis in reoperations.

## Introduction

Facial nerve preservation in parotid surgery was first described in 1907 by Thomas Carwardine (1). It presents a major challenge given the risk of aesthetic and functional damage.

Postoperative facial palsy (FP) is reported in literature at a frequency of approximately 20% for transient FP and 0 to 14% for definitive damage (2-4). The factors that increase this risk are large tumor size, deep location, malignancy, and reoperations (1,3).

Facial nerve monitoring (FNM) was created in the early 1990s (5). However, to our knowledge, there are limited data on the subject found in literature and there are no guidelines for clinical use (6). The benefits and indications of FNM remain to be defined. 

The aim of our study was to assess the use of FNM for the functional preservation of the facial nerve in parotidectomies.

## Materials and Methods

This was a retrospective two-center study of patients presenting to Val de Grâce Military Training Hospital and Percy Military Training Hospital (Paris, France) with parotid tumors from January 2007 to January 2012.

The study population was divided into the following two groups based on the use of facial nerve monitoring: group 1, no FNM (before March 2009, the date of NIM acquisition at the two hospitals) and group 2, FNM.

Facial nerve monitoring was performed using the NIM-Response 2.0 from Medtronic Xomed.

All patients with an indication for parotidectomy were included in this study; they were examined by the ENT service preoperatively and reviewed postopera- tively. All of the patients were operated on by two senior surgeons. The primary endpoint was the House-Brackmann classification at one month and six months postoperatively. Facial palsy was determined when the House-Brackmann grade was 3 or higher. Facial palsy was considered definitive after 6 months. For patients with a partial dissection of the facial nerve, especially cases involving reoperation and partial FP, only total palsy of the involved territory was taken into account. We excluded from our study patients with minimal damage of a distal branch of the facial nerve. They were considered free of facial palsy. For patients with reoperation, the parotid was excised with complete nerve re-dissection. The size and location of the tumor, determined with MRI, and the final tumor histology were also recorded for each of the 2 groups.

A comparative analysis using SPSS for Windows 10.0 statistical software was performed. Student’s t-test was used to compare quantitative data and the chi-square test was used to compare qualitative data. The differences were considered significant at a p-value less than or equal to 0.05.

The hospital ethics committee exempted this study from the need for consent because it only involved retrieving data from medical records (Scientific Committee for Clinical Trials of the Percy Hospital, September 2007).

## Results

Overall, 128 parotidectomies were performed on 125 patients. Forty-seven cases did not undergo FNM (group 1) and 81 cases included FNM (group 2).

The average tumor size was 3.7 +/- 1.6 cm for the entire population, 3.6 cm in group 1 and 3.8 cm in group 2. The tumors were histologically benign in 102 cases and malignant in 23 cases. The differences between the two groups in terms of tumor size, histology, age, and gender were not statistically significant. In group 1 (n=47), 29 superficial parotidectomies, 10 total parotidectomies, and 8 reoperations were performed (Table 1). In this group, 15 FPs were noted; 8 were transient and 7 were definitive (Tables. 1,2).

Among the 29 superficial parotidec- tomies, seven FPs were noted: six were transient and one was definitive. Among the 10 total parotidectomies, four FPs were noted: two were transient and two were definitive. Among the eight reoperations, four FPs were noted, all of which were definitive (Tables 1,2). Two of these FPs represented complete palsies of the *marginal* mandibular branch of the facial nerve caused by partial dissection of the facial nerve (there were no dissections of the upper branches).

In group 2(n=81), 56 superficial parotidec- tomies, 15 total parotidectomies, and 10 reoperations were performed (Table.1).In this group, 19 FPs were noted: 12 were transient and seven were definitive.

Among the 56 superficial parotidectomies, 12 FPs were noted: 10 were transient and two were definitive. Among the 15 total parotidectomies, five FPs were noted: two were transient and three were definitive. Among the 10 reoperations, two FPs were noted, all of which were definitive(Tables 1,2). Significant differences between the two groups were only found with regard to reoperations. The FP rates at one month and six months were significantly higher in group 1 (without FNM) than in group 2 (with FNM) after reoperations.

The differences between the two groups were not statistically significant in cases of first-line surgery.

**Table 1:** rate of postoperative facial palsy to one month

**Type of parotidectomy**	**Group 1** **(n=47)**	**Group 2** **(n=81)**	**P**
Superficial (n=85)	7/29	12/56	07
Total (n=25)	4/10	5/15	08
Reoperation (n=18)	4/8	2/10	035

**Table 2:** rate of postoperative facial palsy to six months

**Type of parotidectomy**	**Groupe1** **(n=47)**	**Group 2** **(n=81)**	**P**
Superficial (n=85)	1/29	2/56	06
Total (n=25)	2/10	3/15	06
Reoperation (n=18)	4/8	2/10	03

## Discussion

According to our results, FNM is only useful during reoperations. It does not improve the facial prognosis in routine procedures, regardless of whether they are superficial or total parotidectomies.

However, these results depend on many factors related to the surgeon’s technical skills, the indication for use, and the expectations of FNM.

*1. Who uses FNM?*

Most head and neck surgeons in Western Europe and the United States of America use FNM during parotidectomies (2). Young surgeons are particularly likely to use FNM because they have been trained in this procedure and have little experience performing parotidectomies without FNM. Surgeons with a history of lawsuits concerning postoperative FP are more likely to use FNM (2).

*2. When to use FNM?*

FNM can be used for any parotidectomy independent of the histology, size, and location of the tumor (2). However, no studies provide guidelines for its use (6). FNM allows younger surgeons to improve their technical skills more quickly and allows experienced surgeons to develop and mentor junior surgeons more easily. 

The systematic use of FNM for all parotidectomies allows surgeons to become familiar with this tool. Thus, during reoperations or surgeries involving large tumors that alter anatomical landmarks, the surgeon will have a high degree of confidence in the use of FNM.

*3.* *The benefits of FNM use during parotidectomies*

3.1 Improved postoperative facial nerve function.

FNM makes it easier to recognize the facial nerve and its branches with minimal traumatic manipulation (2,4). 

The benefits of FNM use with routine procedures to treat benign tumors is controversial. Most studies have shown no improvement in postoperative facial nerve function as a result of these surgeries (1-3,7-12). Our series is consistent with the data reported in literature. For the first-line surgery cases, there was no significant difference between groups 1 and 2. Only two studies have shown a significant reduction in the rate of transient postoperative FP as a result of FNM use (4,13). Only one study has shown a significant reduction in the rate of definitive postoperative FP (13). 

In our series, the use of FNM resulted in a significant reduction in the rate of transient and definitive FP in reoperation cases (P< .05). These results are consistent with the data reported in *literature *(2,11,14,15). These reoperations involve a total parotidectomy when a previous superficial parotidectomy had been performed. The final histology was either malignant or a recurrence of a benign or malignant tumor. In such cases, the anatomical landmarks have completely changed. The fibrous remodeling of the surgical site makes surgery more challenging and FNM is important in these situations (11).

*3.2 Improved surgical procedures.*

A parotidectomy is more difficult to perform when the tumor is large or when it involves cancer. The size of our series did not allow us to detect differences among subgroups. The importance of FNM for the functional preservation of the facial nerve in cases of large tumors and malignant parotid surgeries could not be assessed. 

Fakhry et al. described a case of a large parotid tumor (16). They concluded that in this difficult case, FNM was very helpful for identifying and protecting the facial nerve. However, there are no data in the literature supporting the use of FNM in cases of parotid cancer.

3.3 Reduced the operating time.

The ability to recognize the facial nerve and its branches before cutting fibrous or glandular tissue would reduce operative time (7,9,15).

*3.4 Improved surgeon comfort.*

The risk of postoperative FP depends on the surgeon's experience (17).Using FNM can reassure the surgeon during dissection and thereby reduce nervous tension (2).

*3.5 Facilitation of junior surgeon training at university hospitals.*

FNM is particularly important in university hospitals because it allows junior surgeons to approach facial nerve dissection with speed and greater calm (7,8). It also allows teachers to guide the junior surgeons and to control the surgeon's actions reliably and objectively*.*

*3.6 Checking the integrity of the facial nerve after surgery.*

After surgery, facial nerve trunk stimulation and the visualization of facial responses in 4 fields allow the surgeon to check the electrical activity of the facial nerve. Mamelle et al. found that the post-dissection to pre-dissection ratio of maximal response amplitude is a good predictor of facial function integrity (18). This parameter also allows the surgeon to reassure patients with postoperative FP about the likelihood of recovering facial function (2). From a forensic perspective, these data are interesting.

*4. The risks of FNM*

4.1 False positives and false negatives. 

A false positive signal is one that may result in the misrecognition of the facial nerve. False positive signals are most often encountered upon the distribution of electrical stimulation. Such signals should not occur with well-adjusted monitoring parameters unless the surgeon applies significant traction to the nerve.

False negative signals are a concern because they can result in the non-recognition of the facial nerve. These signals can be explained by the persistence of a fibrous layer that is in contact with the nerve. This relationship emphasizes the importance of careful dissection of branches of the nerve downstream of the stimulation that have been damaged by the tumor, a surgical procedure or, less frequently, by improper adjustment of the parameters.

In their series, Meier et al. described a large number of false positive and false negative signals. They concluded that FNM is not a substitute for knowledge of the anatomy and careful dissection of the nerve during parotidectomies (14). Witt et al. also stated that monitoring can lead to overconfidence, which can give a false sense of security and result in less caution when performing surgical procedures (19).

To prevent this problem, the surgeon should always maintain a critical approach to intervention and monitoring. This approach ensures that monitoring will be used efficiently and rationally.

*4.2 Injuries related to the electrodes.*

Some cases of transient lesions (hematomas, muscle, and eye injuries) have been described (2). Three cases of severe facial burns have been reported following the use of the FNM for parotidectomy. These burns resulted from technical defects in an earlier monitoring model that were likely caused by an electrolysis phenomenon (20). The burns occurred at the level of the electrode insertion and the extent of the burns was related to the duration of monitoring. This phenomenon should not occur when current systems are used. Finally, the benefits of FNM offset the significantly higher risk of skin burns (20).

*4.3 Additional costs of materials.*

Monitoring requires an initial expense because a console must be purchased; however, this cost is offset quickly because the console will be used frequently during otological and thyroid surgery. There are also repeated costs for each intervention related to the use of disposable components (including the electrodes).

Terrell et al. estimated the cost at nearly 380 USD. Therefore, FNM is a useful and inexpensive aid for parotid surgery (4).

## Conclusion

FNM is a simple and effective adjunct method that is available to surgeons during parotid surgery to assist them with the functional preservation of the facial nerve. 

Our results indicate improved preservation of facial nerve function at one month and six months after reoperations that used FNM. However, FNM use did not improve the facial prognosis in first-line surgery cases.

The utility of FNM for malignant and large tumors remains unproven. It would be interesting to continue our series with an increase in sample size and thus statistical power to allow the detection of differences among the different subgroups.
